# Research progress on ferroptosis in Myelodysplastic syndromes

**DOI:** 10.3389/fphar.2025.1561072

**Published:** 2025-02-28

**Authors:** Yifan Yang, Jiongping Han, Yuxin Wei, Jiacheng Jin, Weiying Feng

**Affiliations:** 1 School of Medicine, Shaoxing University, Shaoxing, Zhejing, China; 2 Department of Hematology, Shaoxing People’s Hospital, Affiliated First Hospital of Shaoxing University, Shaoxing, China

**Keywords:** Myelodysplastic syndromes, ferroptosis, signal path, ferroptosis-related genes, ferroptosis inducing drugs

## Abstract

Myelodysplastic syndromes (MDS) are a group of malignancies characterized by clonal proliferation of hematopoietic stem cells, ineffective hematopoiesis, peripheral cytopenias, and a high risk of transformation to acute myeloid leukemia. Current therapeutic strategies for MDS have limited efficacy. Thus, identifying new therapeutic targets and prognostic biomarkers is a critical future research direction. Ferroptosis, a new type of iron-dependent programmed cell death, has become a recent hotspot in the field of oncology research. Recent results have demonstrated that iron metabolism, lipid metabolism, and other pathways can be targeted to induce ferroptosis in MDS cells. In addition, ferroptosis-related genes are of significance in the prognosis and diagnosis of MDS. This article reviews the current research progress on ferroptosis in MDS, including its potential for targeting as a therapeutic intervention strategy.

## 1 Introduction

Myelodysplastic syndromes (MDS) are a group of clonal hematopoietic malignancies characterized by morphological dysplasia of bone marrow cells, as well as anemia, neutropenia, or thrombocytopenia (Arber et al., 2016). The annual incidence of MDS is approximately 4 cases per 100,000 (Sekeres and Taylor, 2022), with higher incidence among elderly patients (Li et al., 2022). Treatment options for MDS are limited, in part because its pathogenic mechanisms remain incompletely elucidated. Currently, the main treatments include supportive care, such as blood transfusions, demethylating agents, chemotherapy, and hematopoietic stem cell transplantation (Cazzola, 2020). However, demethylating agents and other pharmacological treatments have suboptimal efficacy. Currently, hematopoietic stem cell transplantation is the only potentially curative therapy, but due to the median age of MDS patients (around 70 years), stem cell transplantation is usually considered unsafe or impractical in the elderly. Therefore, there is an urgent need to investigate the pathogenesis of MDS and develop new therapeutic approaches.

Ferroptosis, a novel form of cell death that is distinct from apoptosis, autophagy, and necrosis; is triggered by the accumulation of iron-dependent lipid peroxides; and is regulated by various cellular metabolic pathways, including redox homeostasis, iron metabolism, and the metabolism of amino acids, lipids, and glucose (Dixon et al., 2012; Galy et al., 2024; Conrad and Pratt, 2019; Ursini and Maiorino, 2020; Conrad et al., 2018). Studies have shown that ferroptosis induction in tumor cells exhibits anticancer potential in various malignant tumors (Lei et al., 2024). Tumor cells that are resistant to conventional treatments may be more sensitive to this form of death due to imbalances in the lipid peroxidation system. In the field of MDS research, recent studies have shown that ferroptosis signaling pathways regulate the progression of MDS, which suggests that ferroptosis-targeting drugs hold promise as a new MDS therapy approach (Suarez and Gore, 2013). Furthermore, the specific value of ferroptosis-related genes (FRGs) in the diagnosis and prognosis of MDS has been considered. This article summarizes the current domestic and international research progress on ferroptosis in the field of MDS.

## 2 overview of ferroptosis

Ferroptosis is a form of cell death induced by the accumulation of iron-dependent lipid peroxides. Its key processes include abnormal iron metabolism, lipid ROS generation, dysregulation of the antioxidant system, and accumulation of lipid peroxides (Jiang et al., 2021). Ferroptosis is distinct from traditional forms of cell death such as apoptosis, necrosis, and pyroptosis, and it has unique biological characteristics and regulatory mechanisms. Therefore, therapeutic targeting of ferroptosis has been studied as an intervention approach for various diseases, including cancer.

Accumulating research has elucidated regulatory mechanisms of ferroptosis, providing new perspectives and research directions for preventing the occurrence and development of various diseases. In radiation-induced heart disease, total extracts from A. manihot (L.) have been demonstrated to prevent ferroptosis in cardiomyocytes by regulating the NOX4/xCT/GPX4 axis to inhibit redox reactions (Xu et al., 2024a). Furthermore, in calcific aortic valve disease, Nesfatin-1 has been demonstrated to inhibit ferroptosis in aortic valve interstitial cells by regulating the GSH/GPX4 and ZIP8/SOD2 axes (Wang et al., 2024). Similarly, in repetitive traumatic brain injury, SCH79797 inhibits neuronal ferroptosis and reduces NLR family pyrin domain containing 3 (NLRP3) inflammasome activation by promoting PPAR-γ/Nrf2-mediated antioxidant responses (El-Gazar et al., 2024). Each of these studies support the use of ferroptosis-related pathways as potential targets for disease prevention or treatment.

Studies on ferroptosis in tumors are also becoming more widespread (Zhou et al., 2024). In liver cancer, serine beta-lactamase-like protein inhibits the ferroptosis of hepatocellular carcinoma cells by regulating the p53/HSPA8 axis (Zeng et al., 2024); and aristolochic acids suppress ferroptosis via modulation of the p53/GADD45A/NRF2/SLC7A11 pathway (Hou et al., 2024). In non-small cell lung cancer (NSCLC), targeting and regulation of the NRF2/PHKG2 axis promotes ferritinophagy, increases intracellular iron levels, and enhances the radiosensitivity of NSCLC cells through mitochondrial stress-dependent ferroptosis (Han et al., 2024). Likewise, the EGR1/miR-139/NRF2 axis plays a role in ionizing radiation-induced ferroptosis in NSCLC cells (Zhang L. et al., 2024a). In breast cancer, dihydroartemisinin enhances the radiosensitivity of breast cancer cells by inducing ferroptosis *via* the hsa_circ_0001610/miR-139-5p/SLC7A11 pathway (Zhang Y. et al., 2024b). Furthermore, overexpression of hypoxia-inducible factor-1α (HIF1α) increases the sensitivity to Adriamycin and inhibit the proliferation and invasion abilities of breast cancer cells by activating ferroptosis (Yu et al., 2024).

Current research suggests that ferroptosis may also have a key role in hematological diseases. In Acute Myeloid Leukemias (AML), FLT3 inhibitors enhance the sensitivity of FLT3-mutant AML cells to lipid oxidative stress by inhibiting the C/EBPα/SCD axis, thereby inducing ferroptosis (Sabatier et al., 2023). Furthermore, Imetelstat, a first-in-class telomerase inhibitor with clinical efficacy in myelofibrosis and MDS, has been demonstrated to induce ferroptosis in AML cells by promoting the formation of polyunsaturated fatty acid-containing phospholipids, leading to excessive lipid peroxidation and oxidative stress (Bruedigam et al., 2024). In lymphoma, 7-Dehydrocholesterol, an endogenous metabolite, enhances the survival ability of lymphoma cells by protecting their lipids from peroxidation and reducing their sensitivity to ferroptosis, especially for DHCR7-mutated Burkitt lymphoma (Freitas et al., 2024). BRD4 is a bromodomain and extra-terminal domain (BET) protein that positively regulates the expression of ferroptosis suppressor protein 1 (FSP1). BET inhibitors have been shown to reduce the antioxidant capacity within GCB subtype Diffuse Large B-cell Lymphoma cells by decreasing FSP1 expression, thereby increasing their sensitivity to ferroptosis. Moreover, BET inhibitors affect the expression of ferroptosis-related genes, such as SLC7A11 (Schmitt et al., 2023). In Multiple Myeloma (MM), the loss of leukocyte immunoglobulin-like receptor B1 reduces the uptake of LDL/cholesterol by MM cells but activates the cholesterol synthesis pathway to maintain intracellular cholesterol levels. A key intermediate in this pathway is squalene, an effective antioxidant that protects cells from lipid peroxidation damage; cholesterol synthesis pathway activation leads to decreased squalene levels, which causes cells to become more susceptible to ferroptosis (Xian et al., 2024). Additionally, AP-1 inhibitor (T-5224) induces ferroptosis in MM cells by inhibiting the PI3K/AKT signaling pathway (Tang S. et al., 2024a).

## 3 Regulatory mechanisms of ferroptosis in MDS

**FIGURE 1:**
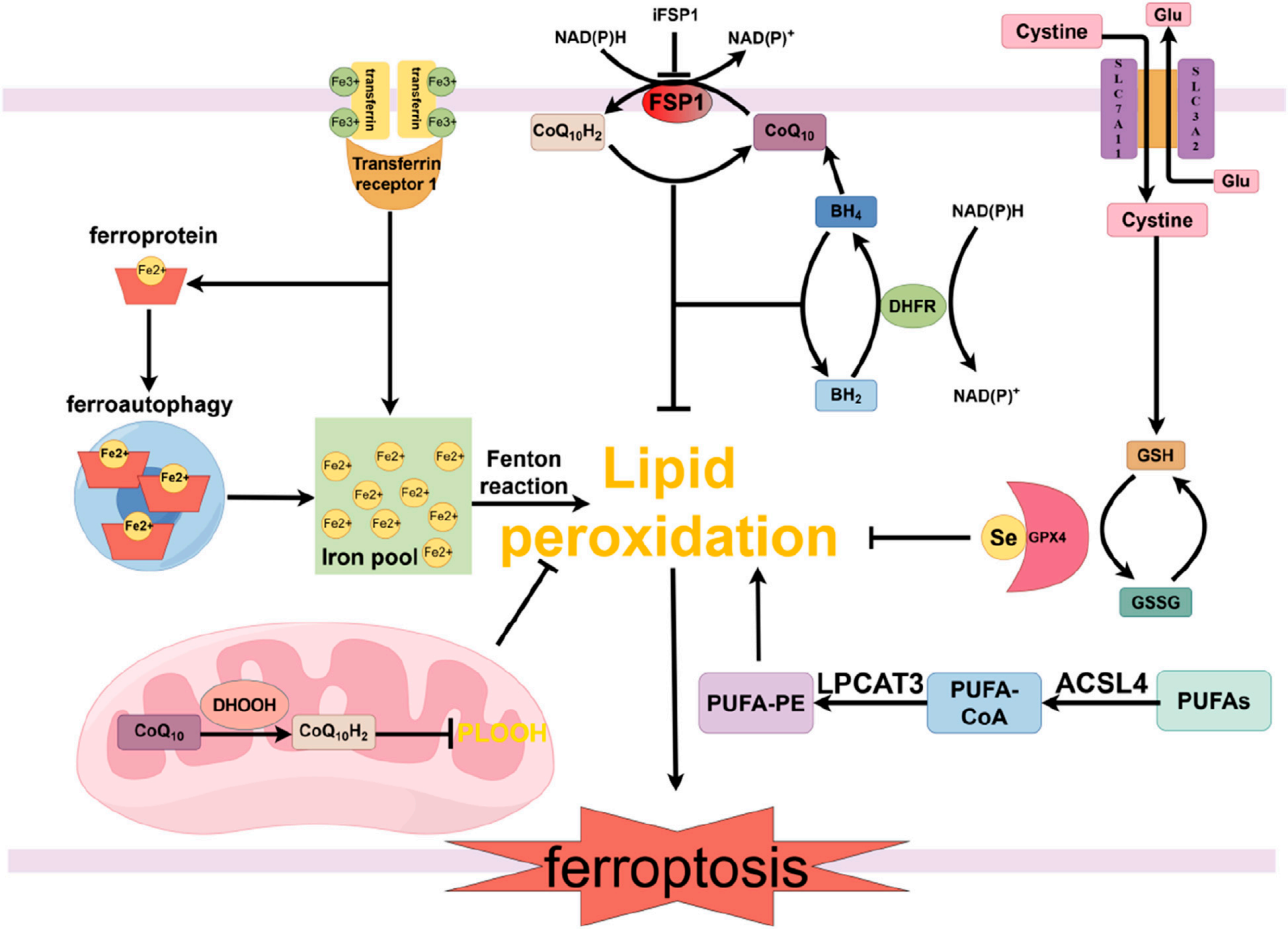
Main molecular mechanisms and signaling regulation of ferroptosis. The mechanisms and related signaling pathways of ferroptosis mainly include iron metabolism disorders, imbalance of the amino acid antioxidant system, accumulation of lipid peroxides, and related signaling pathways mediated by FSP1/CoQ10, GCH1/BH4, and DHODH. GPX4, glutathione peroxidase 4; Se, selenium; Glu, glutamate; SLC7A11, solute carrier family 7 member 11; SLC3A2, solute carrier family 3 member 2; GSH, glutathione; GSSG, oxidized glutathione; PUFAs, polyunsaturated fatty acids; ACSL4, acyl-coenzyme A synthetase long chain family member 4; CoA, coenzyme A; LPCAT3, lysophosphatidylcholine acyltrans-ferase 3; PUFA-PE, polyunsaturated fatty acid-phosphatidyl ethanolamine; CoQ_10_, ubiquinone; CoQ_10_H_2_, ubiquinol; DHODH, dihydroorotate dehydrogenase; FSP1, ferroptosis suppressor protein 1; BH4, tetrahydrobiopterin; BH2, dihydrobiopterin; DHFR, dihydrofolate reductase; FSP1, ferroptosis suppressor protein 1.

As a hematological malignancy, MDS has been the focus of studies on apoptosis (Lambert et al., 2016; Economopoulou et al., 2010; Lefèvre et al., 2017; Czibere et al., 2006; Jing et al., 2024; Liu et al., 2024; McBride et al., 2019; Rivella, 2015) and autophagy (Alexander et al., 2011; Zha et al., 2021; Jacquel et al., 2018; Ames et al., 2023; Weber et al., 2020), but the role of ferroptosis in MDS is not well characterized. Nevertheless, accumulating evidence is consistent with the role of ferroptosis in MDS (Figure 1). Increased Fe^3+^ promotes the generation of ROS (Callens et al., 2010), and iron overload is not uncommon during MDS (An et al., 2023). MDS cells show increased Fe^3+^ uptake and decreased Fe^3+^ efflux, with varying degrees of abnormalities in transferrin, transferrin receptors, and iron metabolism-related proteins. For example, one study demonstrated increased levels of Fe^2+^and elevated expression of transferrin receptor mRNA in CD33^+^ cells of MDS patients (Li J. et al., 2024a). Repeated blood transfusions to improve anemia during treatment is the main cause of iron overload (Pullarkat, 2009; Lindsey and Alex, 2018). Intrinsic ineffective erythropoiesis, a form of hemolysis caused by chemotherapy and hematopoietic stem cell transplantation, further exacerbates iron overload. Additionally, in MDS patients, the activation of NLRP3 inflammasomes is a redox-dependent, hallmark feature that leads to clonal expansion and pyroptosis (Sallman and List, 2019; Sallman et al., 2016), which results in increased ROS levels (Montalban-Bravo et al., 2020; Grignano et al., 2020). Molecular processes related to MDS, such as mutations in NLRP3, as well as drugs like decitabine, affect the production of ROS (Lv et al., 2020). The high ROS state makes MDS cells more prone to ferroptosis. Therefore, inducing ferroptosis in MDS cells by increasing cellular ROS levels is a potentially beneficial approach for treating MDS.

**FIGURE 2:**
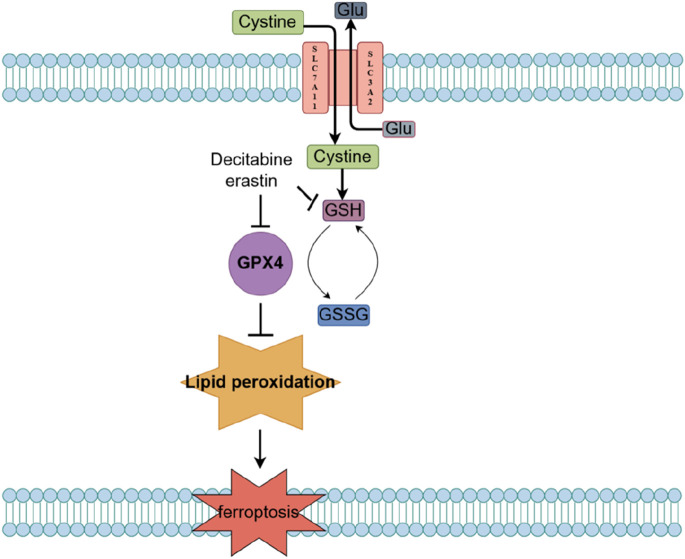
Mechanisms of ferroptosis in MDS.

Current evidence suggests that MDS cell ferroptosis is induced through the System Xc - glutathione (GSH) - glutathione peroxidase 4 (GPX4) pathway (Figure 2). The System Xc^−^-GSH-GPX4 pathway is a classic regulatory pathway of ferroptosis. Within this pathway, System Xc-is a cystine/glutamate antiporter on the cell membrane, responsible for transporting extracellular cystine into the cell to synthesize GSH; GSH is an important intracellular antioxidant that maintains the cellular redox balance; and GPX4 is a GSH-dependent antioxidant enzyme that reduces lipid peroxides on the cell membrane, preventing lipid peroxidation damage. When the System Xc--GSH-GPX4 pathway is inhibited, intracellular GSH synthesis decreases and GPX4 activity is reduced, leading to the accumulation of lipid peroxides on the cell membrane. Excess lipid peroxides cause damage to the cell membrane, ultimately inducing ferroptosis. In support of the role of this pathway in MDS, treatment of the MDS cell line SKM-1 and two myeloid leukemia cell lines (KG-1 and K562) with the ferroptosis inducer erastin was demonstrated to induce ferroptosis by depleting GSH and reducing GPX4 activity.

Decitabine, a natural adenosine analogue of 2′-deoxycytidine nucleotide that is classified as an S-phase cell cycle-specific drug, exerts demethylation by inhibiting DNA methyltransferase, which activates tumor suppressor genes and inhibits cancer cell proliferation; thus, decitibine has been employed in the treatment of MDS and AML (Suarez and Gore, 2013). Notably, decitabine can significantly increase ROS levels and promote the release of iron ions in MDS cells, which further generates reactive oxygen species through the Fenton reaction, thus exacerbating oxidative stress; as a consequence, decitabine reduces GSH levels and inhibits GPX4 activity, leading to the accumulation of lipid peroxides. These findings are consistent with the possibility that ferroptosis may be the main mechanism by which decitabine induces death in MDS cells.

## 4 Prognostic studies of ferroptosis-related genes (FRGs) in MDS

Multiple genes are involved in the regulation of ferroptosis (Su et al., 2024). GPX4 is an inhibitor of ferroptosis that prevents cell death by reducing phospholipid peroxides (Seiler et al., 2008; Roveri et al., 1994; Mishima et al., 2023; Mao et al., 2021; Friedmann Angeli et al., 2014). Moreover, SLC7A11 indirectly inhibits ferroptosis by promoting glutathione synthesis (Xu et al., 2019), while FSP 1 inhibits ferroptosis through coenzyme Q10 (Doll et al., 2019; Bersuker et al., 2019). Additionally, GCH1 inhibits ferroptosis through antioxidant mechanisms (Kishi et al., 2012; Ahn et al., 2023). Conversely, ACSL4 (Ding et al., 2023), ALOX15 (Sui et al., 2024), and NCOA4 (Xu M. et al., 2024) enhance ferroptosis sensitivity by promoting lipid peroxidation and iron ion accumulation via parallel pathways, while TFRC promotes iron ion uptake, increasing the risk of ferroptosis.

With increasing research on ferroptosis genes, the roles of FRGs in hematological diseases are gradually being uncovered. For example, a prognostic model including six FRGs (VEGFA, KLHL24, ATG3, EIF2AK4, IDH1, HSPB1) can optimize risk stratification for AML patients (Jiang et al., 2023). In lymphoma, a risk scoring model containing 16 survival-related FRGs (DRD4, TFAP2C, AKR1C3, CHAC1, ULK2, CXCL2, GABARAPL1, TRIB3, CYBB, IREB2, EPAS1, MT1G, ATG3, ATF4, CAPG, UBC) shows good efficacy in predicting the survival of DLBCL patients (Xiong et al., 2022). In MM, a risk scoring model including five FRGs (YY1AP1, AURKA, CDKN1A, RRM2, STEAP3) can accurately predict the prognosis of MM patients (Wang et al., 2023).

Ferroptosis is also a promising target for MDS therapy, and specific roles of FRGs in MDS diagnosis have been proposed. Using MDS-related microarray data and clinical information from the Gene Expression Omnibus (GEO), the predictive ability of FRGs was evaluated using nomogram analysis and external datasets. A set of six feature genes (SREBF1, PTPN6, PARP9, MAP3K11, MDM4, EZH2) demonstrated high accuracy in MDS diagnosis. These findings underscore the complex relationship between FRGs and MDS (Zhu et al., 2024).

In an alternate study, another group of FRGs (BNIP3, MDM2, and RRM2) were demonstrated to serve as biomarkers for the diagnosis, treatment, and prognosis of MDS. Researchers identified these FRGs using RNA sequencing data and clinical information from GEO, extracting fFRGs from the FerrDb website, and performing differential expression analysis using the R package. Subsequently, Kaplan-Meier and Cox regression analyses were employed to assess the prognostic roles of these three genes. The diagnostic and prognostic efficacy of these genes in MDS was confirmed through Receiver Operating Characteristic curve analysis. Although this prognostic model constructed solely with BNIP3, MDM2, and RRM2 is relatively one-sided, the findings deepen the understanding of MDS pathogenesis, improve risk stratification, and build a more precise MDS prognostic system (Chen et al., 2023).

## 5 Research progress on targeting ferroptosis in MDS

Targeting ferroptosis is of importance in tumor therapy, with potential to provide new strategies for refractory and drug-resistant tumors. Recent studies have shown that targeting of ferroptosis may also provide therapeutic benefits to MDS patients by overcoming resistance to traditional therapies and reducing side effects on normal cells due to its ability to selectively induce death in MDS cells. Additionally, ferroptosis induction can function synergistically with existing treatments by enhancing antitumor effects and shows broad clinical prospects. Three such synergistic strategies are presented below.

### 5.1 Ferroptosis induction combined with cytotoxic chemotherapy

Erastin is a small-molecule compound that promotes the accumulation of lipid peroxides by inhibiting the Xc^−^ system and reducing GSH production, ultimately inducing ferroptosis in MDS cells (Yagoda et al., 2007). Cytarabine is a key chemotherapeutic drug used to treat hematological malignancies such as MDS and AML; it is a cell cycle-specific antimetabolite drug that mainly inhibits DNA synthesis to suppress tumor cell proliferation and survival (Magina et al., 2017; Löwenberg, 2013). Studies have shown that erastin-induced ferroptosis can enhance the sensitivity of MDS cells to cytarabine.

### 5.2 Ferroptosis induction combined with demethylating agents

Decitabine is an antimetabolite drug classified as a demethylating agent; it inhibits tumor cell proliferation by altering DNA methylation status and restoring tumor suppressor gene expression. Studies have shown that when the ferroptosis inducer erastin is used in combination with decitabine, it can further reduce GSH levels and enhance the toxic effects of decitabine on MDS cells.

### 5.3 Ferroptosis induction combined with cuproptosis

Cuproptosis is a novel form of cell death induced by various copper ion carrier drugs such as Elesclomol, Disulfiram, and NSC319726 (Tang D. et al., 2024). Studies have shown that targeting ferroptosis and cuproptosis by inhibiting the xCT-GSH-GPX4 pathway can synergistically enhance the effect of DSF/Cu in MDS treatment, providing insights for this combined therapeutic strategies (Li H. et al., 2024). Other research results indicate that the combined use of ES-Cu and IKE has synergistic effects in MDS treatment, enhancing therapeutic efficacy by inducing multiple programmed cell death pathways (Gao et al., 2024). In the latter study, the induction of cuproptosis and ferroptosis in MDS cells by ES-Cu/IKE was enhanced by modulating the xCT-GSH-GPX4 axis, providing new strategies for MDS treatment.

## 6 Summary and outlook

In conclusion, ferroptosis has a close relationship with MDS. Ferroptosis induction in MDS cells via ferroptosis-related signaling pathways, or in combination with existing cytotoxic chemotherapeutic drugs and demethylating agents, may provide new directions in the field of MDS research. Current research on ferroptosis in MDS is mostly at the *in vitro* and animal model stages and has not yet expanded to clinical studies in patients. Therefore, transforming the basic research of MDS treatment with ferroptosis regulators into clinical practice is a key area for future exploration. The use of FRGs to stratify and evaluate prognosis may facilitate the precise diagnosis and treatment of MDS patients. Because research on ferroptosis in MDS has just begun, its mechanisms of pathogenesis, diagnosis, treatment, and prognosis require further exploration.
